# Developing a conceptual framework for an evaluation system for the NIAID HIV/AIDS clinical trials networks

**DOI:** 10.1186/1478-4505-7-12

**Published:** 2009-05-21

**Authors:** Jonathan M Kagan, Mary Kane, Kathleen M Quinlan, Scott Rosas, William MK Trochim

**Affiliations:** 1 Division of Clinical Research, NIAID/NIH, 6700 B Rockledge Drive, Room 1102, MSC 7609, Bethesda, Maryland 20892-7609, USA; 2 Concept Systems, Inc, 136 East State St, Ithaca, New York 14850, USA; 3 Department of Policy Analysis and Management, Cornell Office for Research on Evaluation, Martha Van Rensselaer Hall, Cornell University, Ithaca, New York 14853-4401, USA

## Abstract

Globally, health research organizations are called upon to re-examine their policies and practices to more efficiently and effectively address current scientific and social needs, as well as increasing public demands for accountability.

Through a case study approach, the authors examine an effort undertaken by the National Institute of Allergy & Infectious Diseases (part of the National Institutes of Health, Department of Health & Human Services, United States Government) to develop an evaluation system for its recently restructured HIV/AIDS clinical trials program. The challenges in designing, operationalizing, and managing global clinical trials programs are considered in the context of large scale scientific research initiatives.

Through a process of extensive stakeholder input, a framework of success factors was developed that enables both a prospective view of the elements that must be addressed in an evaluation of this research and a current state assessment of the extent to which the goals of the restructuring are understood by stakeholders across the DAIDS clinical research networks.

## Introduction

Around the globe, health research organizations are examining ways of designing, managing and evaluating their programs so they address local and international health needs and achieve maximum value for their investments. These practical concerns are sparking interest in research *on *health systems research, in order to improve results and create an evidence base that can guide policy-makers on the management of health research [1].

This manuscript describes the process and progress to date in developing a systematic approach to support the evaluation of a global human immunodeficiency virus (HIV) clinical trials research program. Further, it analyzes and draws inferences from this specific work that may have broad applicability for the management, operationalization, and evaluation of other large-scale research endeavors.

The specific case study highlighted here is situated in the recent restructure of the world's largest human immunodeficiency virus/acquired immunodeficiency syndrome (HIV/AIDS) clinical trials program. We begin by reviewing the program's context, its scientific goals and objectives, rationale for the restructure, and how the factors that drove the restructure were addressed in the restructuring plan. We then focus on the development of a conceptual framework as the foundation of a larger evaluation system, emphasizing the process and results of a stakeholder-driven effort to describe the success factors for the newly restructured program. We discuss the resulting framework in the context of the program's goals and its implications for the evaluation of other large scale research endeavors.

## Background on the transformation of science

In recent years, there has been a broad transformation of the organization and management of science in the United States [2,3]. In addition to traditional support for individual scientists working on their own, federally funded scientific research now emphasizes "big science": large, collaborative research initiatives, with annual budgets of $5 million or more. The number of these types of initiatives has increased in recent years [4].

One major type of "big science" is clinical research networks of which the HIV/AIDS clinical networks described in this manuscript are a prominent example. There are nearly 300 clinical research networks in the United States and Canada. The majority are funded primarily by the US Government and nearly half carry out clinical trials as their primary activity [5]. Non-trial studies include observational research, outcomes research or best practice modeling.

Another type of "big science" is the extramural research center structure, first funded in the 1960s. There are now more than 1200 center programs, with each of the National Institutes of Health (NIH) institutes using this approach [6]. These programs generally take a multi-disciplinary team approach focused on interaction between basic and clinical researchers to foster translational research.

Large research initiatives generally have broader goals than traditional individual investigator-initiated grants [7]. In addition to providing scientific outputs, these multi-million dollar, multi-institutional programs are also expected to foster multi-disciplinary teamwork, provide more effective support for independently funded investigators, gain increased attention to a program's research by the center's home institution, recruit established researchers to the program's area of interest, develop new investigators, expand the education of health professionals and the general public, build scientific infrastructure and demonstrate state-of-the-art prevention, diagnosis and treatment techniques. Ultimately, the efficiencies and synergism inherent in these systems of research are expected to positively impact population health and behavior by producing innovative, relevant, and timely research. These broader goals, and the operational activities that support them, must be taken into account when developing evaluation approaches [3].

Thus, the multiplicity of goals and stakeholder agendas which these research enterprises simultaneously pursue, poses substantial management, implementation and evaluation challenges. At the same time, there are growing pressures for accountability for federally funded scientific research [8-11], and recent law to include performance monitoring officers. [12,13].

Leading scientific bodies, responding to government expectations, have issued their own recommendations for the evaluation of scientific research endeavors [6,14-16]. This paper explores the management and evaluation challenges associated with the HIV/AIDS clinical research networks. Through a case-study approach, we describe a participatory process applied in this setting to construct a conceptual framework, and explore the utility of the framework to shape a system of network evaluation.

## Background on the NIAID Division of AIDS and its clinical trials networks

The Division of Acquired Immunodeficiency Syndrome (DAIDS) is one of three extramural scientific divisions of the National Institute for Allergy and Infectious Diseases (NIAID), the second largest of the 27 institutes and centers that comprise the U.S. National Institutes of Health (NIH). Established in 1986, the mission of DAIDS is to help ensure an end to the HIV/AIDS epidemic by increasing basic knowledge of the pathogenesis and transmission of the human immunodeficiency virus (HIV), supporting the development of therapies for HIV infection and its complications and co-infections, and supporting the development of vaccines and other prevention strategies. With an annual budget of approximately one billion dollars, DAIDS is a major source of funding for biomedical research around the world.

**Figure 1:**
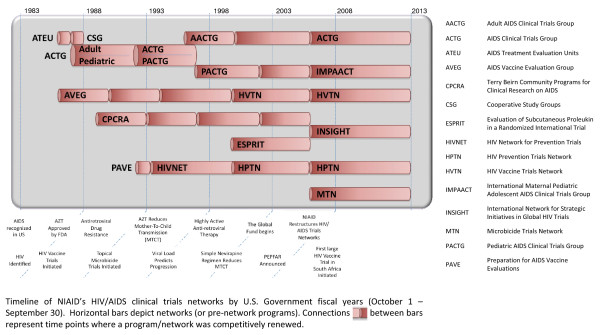
**Evolution of the NIAID HIV/AIDS clinical trials networks**.

Beginning with the AIDS Treatment Evaluation Units in 1987, DAIDS goal in establishing the HIV/AIDS networks was to assemble multidisciplinary scientific expertise, and create sustainable, reusable capacity and infrastructure to support a long-term commitment to clinical trials. The ensuing 19 years witnessed significant scientific advances as well as major changes in the global demographics of HIV/AIDS, presenting both challenges and opportunities for research. In response, the DAIDS networks have evolved and adapted. An overview of the history and evolution of the DAIDS networks is shown in Figure 1.

### Restructuring the HIV/AIDS clinical trials program

In 2001, several factors, including the size and complexity of the network enterprise, changes in the HIV/AIDS knowledge base, unabated expansion of the epidemic in the developing world, and fiscal considerations, prompted the DAIDS leadership to reexamine the organizational and funding structure of the networks. Taking into account these and other factors, the Division began working with investigators, collaborators, advisory committees, community groups, and a wide range of stakeholders to help develop the scientific priorities going forward, and gather input on how best to restructure the clinical trials programs in anticipation of a grant competition in 2005. Six scientific priority areas for HIV/AIDS clinical trials research emerged from this effort:

• HIV Vaccine Research & Development

• Translational Research for Therapeutic Development

• Optimization of Clinical Management

• Microbicide Research & Development

• Mother-to-Child Transmission of HIV

• Prevention Research

Further, the following key guiding principles were identified:

#### Responsiveness

Maintain a flexible and responsive approach to emerging research challenges that ensures that the highest research priorities are addressed

#### Efficiency

Improve efficiency through the shared use of key support services (e.g. laboratories, pharmacies), common data elements and harmonized data systems, coordinated specimen management, shared/standardized training for common needs, coordinated clinical product acquisition, distribution and provision.

#### Coordination

Harmonize and/or integrate HIV/AIDS prevention, vaccine and therapeutic research at the scientific and community levels, in order to maximize research opportunities and better respond to public health needs

#### Capacity Building

Build and strengthen HIV/AIDS research capacity, especially in resource-limited settings, both domestically and internationally

#### Evaluation

Strengthen evaluation of network science and management to help ensure that the networks address the highest priority research questions, and function as efficiently and effectively as possible through the identification and implementation of best practices, and linking funding to performance.

To put these scientific and operational priorities into place, DAIDS developed and issued two "Request for Application" (RFAs) that differed substantially from previous network solicitations in several ways.

1. Whereas in the past DAIDS issued multiple "dedicated" RFAs (one for each clinical trials network it sought to renew), this time a single Leadership Group RFA was used. Competition through a single RFA highlighted the importance of the linkages between the scientific priorities, the applicability of the guiding principles to all applicant networks, and the decision not to set a pre-determined funding level for any one (or group) of the six scientific area(s).

2. Similarly, a single RFA was used to solicit applications for Clinical Trials Units (CTU) seeking funds to carry out the future network trials. In previous funding cycles, DAIDS had issued RFAs for CTU that were 'specific' to a particular network. The rationale behind the single CTU RFA was to maximize scientific opportunity and efficiency by allowing investigators (including those in resource poor settings) interested in participating in more than one type of clinical trial (e.g. vaccines and treatments) to describe their capabilities, and how they would coordinate and harmonize this research, within a single application. This approach also supports DAIDS goal to help build HIV/AIDS clinical research capacity by making grant awards directly to institutions in areas where the disease has hit the hardest, and where resources are often constrained.

3. To stimulate the implementation of a cross-cutting and interdisciplinary research agenda, both RFAs described four key organizational elements/activities: 1) a new committee comprised of the Principal Investigators of the networks; 2) a new central office of HIV/AIDS network coordination; and 3) a new Community Partners group, to enhance community input on all levels, and assure effective representation of (and timely communication among) the many communities, (domestic and international) within which the networks conduct research, and; 4) coordination, communication and collaboration across the Institutes and Centers at NIH that co-sponsor or, in other ways, contribute the HIV/AIDS clinical research networks.

4. To help address the likely scenario of emergent high priority research opportunities that could require timely reordering of scientific priorities, new flexible resource allocations were developed. Both RFAs described the importance of performance evaluation, designation of new 'reserve' funds, and a role for external advisory bodies to assist in meeting such challenges.

In June 2006, NIAID announced funding for the restructured HIV/AIDS clinical trials networks:

• AIDS Clinical Trials Group 

• HIV Prevention Trials Network 

• HIV Vaccine Trials Network 

• International Maternal Pediatric Adolescent AIDS Clinical Trials 

• International Network for Strategic Initiatives in Global HIV Trials 

• Microbicide Trials Network 

Together, including the network leadership groups and 73 CTU located around the world, funding for the networks was approximately $285 million in the first year of awards.

### Evaluation goals for the restructured networks

DAIDS considers a comprehensive, integrated evaluation system a vital element of the new coordinated network structure. Prior to restructuring (and currently), evaluation activities have been almost exclusively 'network centric'. Each group's investigator leadership has primary responsibility for developing and implementing criteria and processes for assessing the structural components *within *a single Network, such as the clinical trials units, laboratories, data management centers, scientific and resource committees, and protocol teams. In some instances, ad hoc expert review panels have provided external input and evaluative information. While such single network-focused evaluation activities remain very important, DAIDS also recognizes the need for a cross-cutting evaluation framework that can support the common guiding principles that span the restructured network enterprise (e.g. responsiveness, efficiency, coordination, capacity building). Such a system can assist DAIDS, its investigators and collaborators in ensuring that the highest priority scientific objectives are addressed; while supporting collaboration, efficiency and research integration at the cross-network level. While the approach being developed is designed to build upon and complement existing within-network evaluation approaches, it will also define criteria and measures that should be applicable across the networks. It will address a variety of factors required for the success of clinical research networks, including activities and processes based within (as well as across) the investigator networks, constituency communities, collaborators (e.g. support contractors), and DAIDS itself (e.g. regulatory, safety, clinical research policy).

The DAIDS HIV/AIDS clinical trials networks are an example of a large scale scientific initiative and as such, share much in common with similar ventures, including broad goals, a multidisciplinary focus, and emphasis on team science. However, the HIV/AIDS clinical trials networks are unique due to aspects related specifically to the conduct of international human clinical research, namely: 1) the reliance on inter-institutional collaboration among multiple clinical research centers and other collaborators (e.g., pharmaceutical sponsors, other co-funding agencies); 2) interaction with research constituents/advocates, including vulnerable populations, and related ethical issues; 3) compliance with regulatory authorities of multiple countries which frequently are not harmonized; 4) the need to maintain a consistent and yet humanitarian distinction between research and care; 5) achieving balance between competing clinical research priorities (e.g. prevention & treatment, domestic & international). Ultimately, DAIDS seeks to support the goals of the clinical research networks, individually and collectively, toward research progress against HIV/AIDS.

**Figure 2:**
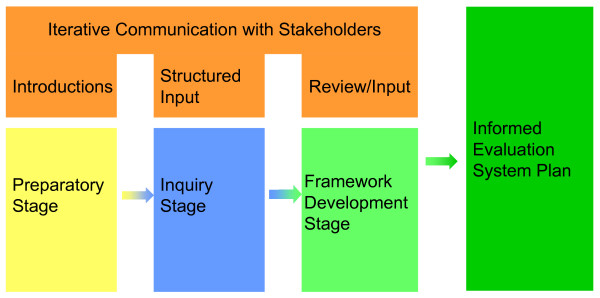
**Process for developing an evaluation system**.

While most program evaluations are ad hoc, this effort is unique in that it focuses on creating a system of evaluation. The development of an evaluation system for the DAIDS clinical research networks encompasses several integrated phases. Figure 2 displays the focus and sequencing of these phases leading to an evaluation system, including preparation, inquiry, framework development, and the evaluation plan. In this paper we describe the initial phases in this process: the creation of a conceptual framework and show how it informs and connects to the other planning steps.

## Engaging stakeholders in constructing an evaluation framework

There are numerous challenges in developing appropriate evaluation systems for large research initiatives. These include: ensuring the highest-quality evidence; minimizing burden and the intrusiveness of the system; and yielding data that can satisfy a myriad of scientific, managerial, financial, and regulatory requirements. One of the most challenging aspects is identifying the goals of a complex research initiative across a wide array of stakeholders, each with a set of expectations as to what constitutes success. There are few precedents to guide evaluation planning for such efforts. With the expectation that today's scientific research enterprise is collaborative and coordinated at multiple levels, several sources of input are needed to define the goals of these complex and diverse systems, develop strategies to work across disciplines in new and innovative ways, and evaluate the outcomes of collaborative work. Due to the broad range of activities, potential outputs, and outcomes of large research initiatives, it is essential that a comprehensive conceptual model to serve as guide for evaluation is developed [17]. Despite some initial work describing the evaluation of science and technology initiatives [6,15,17-21] there is little guidance on methodologies that would be appropriate, and even less experience in implementing them [22]. Moreover, while some have examined the management of clinical trials research [23-25], there is little research on the evaluation of clinical trials research.

Many of the challenges found in evaluating scientific research initiatives can be mitigated by engaging the stakeholders of the system, particularly those not often involved in determining organizational evaluation elements, in the process of evaluation planning. In this section, we describe the approach to engaging stakeholders in the construction of the conceptual framework to guide the development of an evaluation system for the DAIDS clinical research program.

To construct the evaluation framework in an informed way, the engagement of a diverse array of stakeholders was deemed necessary. The project sought input from those who will ultimately be involved in implementing the evaluation, including DAIDS staff, Network investigator leadership, the HIV/AIDS Office of Network Coordination, network evaluation coordinators, community advisory board representatives, and other government agency staff with a role in the networks. Our goal was to achieve a broad sampling of ideas rather than a representative sampling of persons. Thus, the approach was purposive, sampling for heterogeneity. The focus on broad heterogeneous participation was intended to ensure consideration of a wide variety of view points from those intimately familiar with the networks, provide the necessary breadth and depth needed to accurately describe the processes and outcomes of the clinical research networks, and encourage greater buy-in of the results.

The group of stakeholders to inform this initiative was expected to include individuals who:

• Are familiar with and understand clinical trials networks from a practice perspective

• Have familiarity with government research grant-making agencies and mechanisms

• Have current knowledge of HIV AIDS related clinical research

• Understand community perspectives related to HIV AIDS

• Have operational and management responsibility for the Division of AIDS

• Have operational and management responsibility for network(s)

An invitation approach was taken to encourage engagement at al relevant levels within the networks, DAIDS, NIAID and related offices. Within the networks and DAIDS, individuals representing a wide range of positions, job responsibilities and seniority were invited.

The rationale for the involvement of network stakeholders in the conceptual framework is rooted in participatory approaches to evaluation where those with a stake in the program or initiative (partners, funders, beneficiaries) assume an active and substantive role across all phases of the evaluation [26,27]. It is believed that participation in identifying relevant evaluation issues and questions, planning the evaluation design, selecting appropriate measures and data collection methods, and gathering and analyzing data, contribute to enhanced utilization of the resultant evaluation [26,28]. At its core, this participatory evaluation activity provided the opportunity for those with close knowledge of and experience with the DAIDS clinical research enterprise to proactively design how they think the enterprise should be evaluated. This approach has the dual benefit of yielding evaluative information that is useful and meaningful to the people involved in the work, and reduce (or minimize the impact of) the potential imposition of evaluation approaches that are not appropriate to this endeavor or context imposed from outside the initiative. A recent review of a dozen large federal evaluations revealed that the thoroughness of the design process is the most critical factor in successful implementation of a large scale evaluation [29].

Within this context, the key method was a structured group conceptualization process (colloquially referred to as concept mapping) for gathering and organizing input from a wide range of sources. Concept mapping is a well established mixed methods social research approach that integrates rigorous multivariate statistical analyses with familiar qualitative processes such as brainstorming, sorting and rating of ideas to create a shared conceptual model [30,31]. The concept mapping methodology had been used successfully in similar initiatives [17,18,21,32]. While a concept map is not necessary for the development of an evaluation system, the concept mapping methodology offered several advantages for this development process as it presents a rigorous structured approach that can be used effectively by scientists, managers, and community advocates in the process of articulating the conceptual and logical models that underlie the complex, collaborative work within the scientific research enterprise.

The methodology involves several distinct steps. In the first part, the idea generation step, stakeholders were invited to contribute ideas that completed the "focus statement" sentence: "Coordinated clinical research networks will be successful if...". Participants represented DAIDS staff and leadership, representatives of other government agencies, networks, clinical trials units and clinical research sites, the community and other constituencies having an interest in HIV/AIDS clinical trials research. In addition, numerous individual interviews were conducted and analyzed, together with documents related to the networks. The statements submitted in the initial idea generation were then synthesized into a representative set of success factors (eliminating duplicates, ensuring clarity and relevance to the focus statement, achieving a common level of specificity) that participants could work with during the idea structuring phase.

The ideas were then structured [33-35] by having stakeholders sort them into categories of conceptual similarity, and by rating them on their importance to the success of the networks, using a 1 (relatively unimportant) to 5 (extremely important) scale.

Using a series of statistical analyses (multidimensional scaling, then hierarchical cluster analysis), participants' views about how to categorize the success factors were aggregated [33-35]. The result is a single, co-authored conceptual map of the territory that illustrates the relationships between ideas [36-38]. The map clusters organize the input into groups of ideas (major concepts) [39,40]. Participants' ratings of the ideas make it possible to determine priorities, compare the importance of different success factors, assess consensus across groups, and create value maps for specific groups of stakeholders.

## Evaluation framework findings

### Overview of the framework

Over 300 stakeholders, representing every sector of the HIV/AIDS clinical research stakeholder community, contributed ideas that completed the "focus statement" sentence, "Coordinated clinical research networks will be successful if...". More than 1500 ideas were generated in response to that focus statement, which were then synthesized into a representative set of 91 ideas that describe what stakeholders believe are the factors critical to the success of the coordinated clinical research networks. Ninety stakeholders sorted the ideas by similarity/relatedness, and 308 stakeholders rated the ideas with respect to their importance to the success of the research networks. Table 1 summarizes the demographic characteristics of those who completed the ratings.

**Table 1:** Demographic characteristics of raters

Participant Characteristics Questions	Response Choices	Frequency	%
Years Involved	0–5 years	52	17%

	6–10 years	60	19%

	11–15 years	73	24%

	16–20 years	81	26%

	Longer than 20 years	38	12%

	*did not respond*	4	1%

		308	100.00%

Primary Area of Science	Vaccine Research & Development	53	17%

	Translational Research/Drug Development	38	12%

	Optimization of Clinical Management, including Co- Morbidities	112	36%

	Microbicides	17	6%

	Prevention of Mother- to- Child Transmission of HIV	23	7%

	Prevention of HIV Infection	23	7%

	Other/Not applicable	38	12%

	*did not respond*	4	1%

		308	100.00%

Primary Role	Network Core	81	26%

	Clinical Trials Unit/Site	107	35%

	Laboratory	14	5%

	Government	47	15%

	Community	12	4%

	Pharma/Biotech	2	1%

	Pharmacy	2	1%

	Institutional Review Board/Ethics Committee	0	0%

	Non- Governmental Organization	10	3%

	Advisory Group/Committee	6	2%

	Other	23	7%

	*did not respond*	4	1%

		308	100.00%

**Figure 3:**
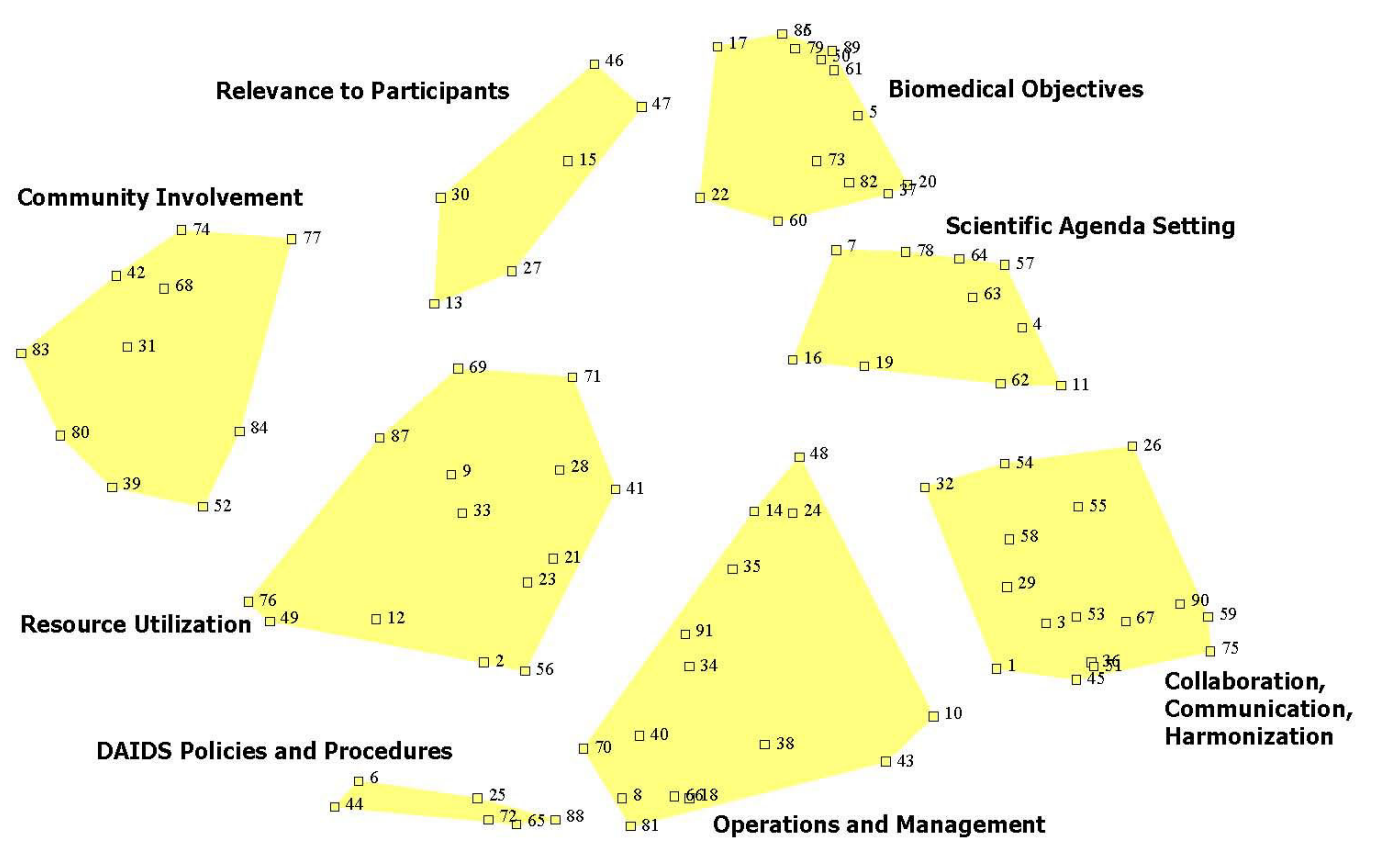
**Concept map of success factors**.

The concept map representing the success factors is shown in Figure 3. On the map, each idea (represented by a point labeled with an identifying number), is shown in relation to all of the other ideas in the framework. Ideas that are closer together were deemed similar (or related) by the individuals who sorted them. In contrast, ideas that are farther apart on the map were judged to be less conceptually similar. The clusters, indicated by the shaded polygons, reveal categories of ideas. Larger clusters signify groups of ideas that are more 'loosely' related, whereas clusters that appear small (or 'tight') indicate groups of ideas that were seen as very closely related.

To facilitate communication about the map and its components, each cluster has been labeled to describe the thematic content of the ideas that comprise the cluster. Just as with the individual ideas (numbered points), clusters that are physically closer together were deemed more similar, according to participants, than those more distant from one another. It does not matter whether an idea appears at the top or bottom of the map; a map could be rotated in any direction, so long as the relationships between the points are preserved. Distance between points – conceptual similarity – is what matters on a concept map. For more information about concept mapping in the context of this project, please see Concept Systems, Inc., 2008 [41].

The evaluation framework depicts the broad range of factors that, in the view of the stakeholders, affect the success of the coordinated clinical research networks. The results illustrate the breadth of the activities and outcomes associated with this type of large scale, collaborative scientific research initiative.

### Analysis and interpretation of the framework

Focusing on each of the clusters within the concept map, and the ideas which comprise them (Additional File 1) allows for a more detailed analysis of the stakeholder-identified success factors in relation to one another and to the goals and guiding principles for the restructured clinical research networks. Additional File 1 shows the individual ideas that make up each cluster. It also presents the average importance rating (on a 1–5 scale) for each of the ideas and the average importance rating of the statements within each cluster. This table allows us to compare the values that stakeholders, as a group, place on broad themes (clusters), as well as individual ideas. Using the ratings to compare subgroups of participants to each other to determine similarity or divergence of opinion on the importance of the items on the map to evaluation, we compared ratings of those who identified themselves as related to different scientific research emphases, (e.g. vaccine research and development, translational research/drug development, optimization of clinical management, and prevention of HIV infection). Overall, we found a great deal of correspondence between different stakeholder groups' average cluster ratings, such that a strong correlation existed from group to group on the conceptual areas that emerged as being of highest importance and those considered less important, indicating similarity in the stakeholder-derived patterns across the elements of the evaluation framework.

Beginning with the cluster labeled *Biomedical Objectives *(situated at approximately "1 o'clock" on the map), this group of ideas focuses on the ultimate aim of the trials networks to identify prevention and treatment strategies for HIV/AIDS that can lead to fewer new infections, and reduced morbidity and mortality in people living with HIV. Of the eight clusters on the map, *Biomedical Objectives *emerged as the most important success factor. That cluster had the highest average importance score (4.11) (Additional File 1) and it includes the highest rated (across all the 91 success factors in the framework) individual statement, "produce high-quality, scientifically valid results" (#82 with a rating of 4.74). Traditional peer review usually places the significance of biomedical research objectives highest, in terms of importance, when evaluating research proposals in the biomedical sciences. That the biomedical objectives of the clinical research networks were seen, across stakeholder groups, as the most important component of success suggests that, at least in this context, large research initiatives do not differ from conventional investigator-initiated research.

Moving "clockwise" on the map, the adjacent cluster, *Scientific Agenda Setting*, is comprised largely of statements that reflect the need for, and the means by which, networks identify their research priorities (Additional File 1). One of the greatest challenges to a cooperative group is to make choices from among an array of competing ideas. In striving to achieve balance in their research agendas, networks are constantly weighing factors such as portfolio diversity, stakeholder input, scientific opportunity, capability and feasibility. Interestingly, both this *Scientific Agenda Setting *cluster, and the *Biomedical Objectives *cluster connect very strongly to one of the most important guiding principles for the restructured networks, namely, responsiveness (Section III A.). That these two clusters were both rated among the top 3 success factors (average importance of 4.06), and were seen as highly related (as indicated by proximity on the map) would appear to affirm a close alignment between DAIDS goals in restructuring the networks, and what the network stakeholders believe most important for success.

Next (again moving clockwise) is a large cluster labeled *Collaboration, Communication and Harmonization*. The emphasis of the ideas in this cluster is, as its name implies, on collaboration and communication both within and between networks. Statements in this cluster (Additional File 1) reference collaboration activities at the scientific level, e.g. "the vision and goals are shared" (#55), as well as at the operational level, e.g. harmonizing key functions like training, laboratory, and data management across networks. The contents of this cluster reflect the restructuring principles of efficiency and coordination, which emphasize an integrated approach to HIV/AIDS vaccine, prevention and therapeutics clinical trials, in order to maximize research opportunities and respond to public health needs in an increasingly efficient manner. The importance of linkages across the scientific priority areas was a key element in the planning process for the restructuring and was the primary impetus for issuing a single network leadership RFA (as opposed to separate RFAs for each clinical research area, as had been done in the past). This was also the major impetus for the establishment of the Office of HIV/AIDS network coordination, formation of a network Principal Investigators committee, the assembly of external scientific advisory bodies to assist with cross-network scientific prioritization, and strengthened coordination across NIH Institutes and Centers. Interestingly, in contrast to the previous clusters, this group of ideas was viewed as being among the lowest in importance among the success factors, with an average rating of 3.81 (keeping in mind that, in this framework, least important does not mean unimportant). Given that many of the statements in the cluster are more process-oriented, it is not surprising that participants view the longer term, ultimate goals of the network (such as in the *Biomedical Objectives *and *Scientific Agenda Setting *clusters) as more important than the activities that lead to these types of outcomes. Also, the activities of resource sharing and inter-network coordination are largely a new demand on the networks, mandated through the restructuring process. While stakeholders recognize this concept as a factor in the success of the networks, these results would suggest that it is not yet fully valued by all participants.

Similarly, *Operations and Management *is also rated relatively lower in importance (3.80) by participants. Again, this cluster pertains to administrative, rather than scientific aspects of the networks' activities. The statements in this cluster also indicate that operations and management depend upon both the networks and DAIDS and suggest that a comprehensive evaluation of the enterprise includes both the grantees and the funding agency, DAIDS.

Attention to funder issues is even more explicit in the *DAIDS Policies and Procedures *cluster. As stated by stakeholders, for the coordinated clinical research networks to reach their goals, DAIDS policies must "reflect what is required for good science, protection of human subjects, and safety", be "transparent", "streamlined" and "clear". This cluster is very much process oriented, and is rated relatively lower in importance compared to other clusters (3.96).

The cluster entitled *Resource Utilization *contains subtopics that, taken together, describe the concept of resources in several ways, including human, time, materials and locations. For example, human resources issues include that "principal investigators demonstrate scientific leadership and innovation" and that they are able to "commit adequate time to network activities." Specific attributes of staff are mentioned, including the need for diversity and expertise in infrastructure development. Other statements focus more on issues related to clinical research site capacity and presumably refer to the many HIV/AIDS trials sites located in developing countries, where access to resources may be limited. Thus, a common theme across both the people and the functions is related to making wise use of resources. This set of statements relates closely to the principle of capacity building that figured prominently in the network restructuring design. The principles of efficiency and coordination also permeate this cluster. Capacity at the site level, in particular, is a focus of existing network evaluation activities. *Resource Utilization *is the fifth most important cluster (out of 8), with an average rating of 4.00.

On the left side of the map, *Community Involvement *emerged as one of the four highest rated success elements in the framework at 4.05. Within this cluster, the idea with the highest importance rating is: "the dignity and human rights of participants are respected." (#42). In addition to the protection of human subjects, the statements in this cluster emphasize the importance of community involvement at all levels and in every stage of study development and implementation. Adequate training and support must also be provided to enable this community involvement. This cluster sheds light on a topic that may be of special significance to clinical trials (by comparison to other types of research). Community involvement plays an essential role in ensuring the ethical and scientific quality of HIV/AIDS clinical research, its relevance to affected communities, and its acceptance by such communities (2007 UNAIDS document *Ethical considerations in HIV preventive vaccine research*). Community involvement may also be more challenging with HIV/AIDS (vs. other diseases) because the communities of interest often reside in resource poor settings (internationally and domestically), the social stigmas attached to HIV/AIDS, and the "disparities in power, wealth, education and literacy which often exist between the individuals proposing to conduct research and those who are hardest hit by the HIV epidemic" [42]. This cluster relates directly to one of the key components of the network restructuring – the new Community Partners organization, whose very purpose is to enhance community input on all levels, and assure effective representation of and timely communication among the many communities involved with network research.

Closely related to the issue of community involvement is *Relevance to Participants*. The focus of this cluster is on ensuring that network research: a) addresses questions of relevance to the communities and trial participants, and; b) can produce results leading to preventions and treatments that can be expected to be made available to the study participants and their communities. Special populations affected by the HIV/AIDS epidemics raise significant ethical considerations, some of which are captured in this cluster. This cluster's location directly adjacent to *Biomedical Objectives *(signifying that stakeholders found these elements to be closely related), is also notable, as this cluster addresses issues regarding who is involved in the studies and who will be the beneficiaries of the results. *Relevance to Participants *emerged as a very important success factor (4.09), second only to the *Biomedical Objectives *of the networks.

The contextual knowledge required to understand or derive value from the concept map can vary depending on the user's focus. For those whose interest is to gain a sophisticated understanding of what the stakeholders of the HIV/AIDS trials networks believe are the factors critical to the success of the venture, it would be important to have: 1) a good familiarity with the NIH's and the investigators' goals for the networks; 2) knowledge of the diversity and multidisciplinarity within the networks; 3) a background in how clinical trials are conducted; 4) a grasp on the global pandemic from many aspects including the epidemiology, demographics, scientific and ethical challenges; 5) an understanding of the special operational and logistical challenges of HIV/AIDS clinical research; and 6) knowledge of the environment/milieu of related/surrounding efforts in which the networks function. With this kind of contextual knowledge, the relationships between individual statements (points) and groups of statements (clusters) on the map would have find their greatest meaning and could potentially provide the most insight as to how the data could be utilized.

## From evaluation framework to evaluation plan

**Figure 4:**
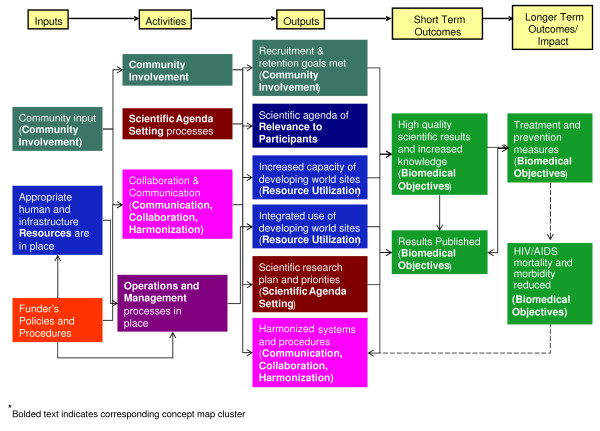
**DAIDS clinical trials networks logic model**.

The concept map of success factors provides a framework upon which subsequent evaluation planning is continuously built. The concept map was used as the basis for a logic model of the coordinated clinical research networks that captures stakeholders' assumptions about how program activities lead to desired outcomes [32,43]. Together, the concept map and logic model form an integrated view that captures the complexity of the research enterprise as well as how it could be evaluated. The concept mapping analysis and results describe the elements of success and their relative importance, and the logic model sequences the elements of success temporally and causally. Graphically, the concept map depicts the multivariate related components of the emerging evaluation framework, allowing the logic model to represent the hypothesized causal pathways between elements identified through the concept mapping process. The current logic model for the DAIDS clinical research networks is depicted in Figure 4.

Typically, logic models are constructed from lists of inputs, activities, outputs, and outcomes, often developed and considered categorically and independent form one another. The spatial features found in the concept map are important because it suggests that those clusters of ideas that are closer are more likely to be connected in the logic model. The understanding of how these concepts are related facilitates a more informed and efficient transition from the map to the logic model. For example, the location of the *DAIDS Policies and Procedures *cluster in relation to the *Biomedical Objectives *cluster suggests they assume opposite ends of the logic model.

Each box in the logic model corresponds to a component or cluster obtained from the concept mapping analysis. The logic model describes a presumed program theory for the complex research initiative and conveys the sequence of expected processes and outcomes. In the end, the logic model developed as a part of this approach was based on a set of concepts that came directly from the stakeholders; with the components of the final logic model directly linked to the original concept mapping ideas submitted by the stakeholders. Thus, the logic model is more representative of the reality of the research networks, than had only one component of the system developed the model. This collaboratively developed model serves as a guide for identifying critical outcome pathways of greatest relevance and for identifying where measurement should be focused as the plan is implemented.

An Evaluation Measurement Task Force (EMTF), consisting of evaluation coordinators from each of the clinical trials networks, along with selected DAIDS staff with evaluation expertise and knowledge of the networks, assisted in interpreting the framework and logic model. The EMTF used the concept map of success factors and the logic model as the foundation upon which to develop proposals for evaluation questions (*what *should be evaluated) and measures (*how *those priority elements should be evaluated), as well as how these efforts should be operationalized in the networks and DAIDS. The EMTF's operationalizing efforts focused on compiling evaluation tools, processes, and methods from networks, developing potential indicators for concepts found in the framework, and identify potential data sources. Thus, the involvement of the EMTF in further interpretation and planning ensured a direct correspondence between the map, content, questions, and measures. This linkage is important as data is collected and analyzed in the future.

The evaluation system design approach being described herein, seeks to incorporate a set of measures that, when taken together, can meet the varied needs of system stakeholders and yield results from which reasonable inferences about performance, across the entire clinical trials enterprise, can be made, including DAIDS, the trials networks, community, clinical research support services, etc. In some areas on the concept map (e.g. Operations and Management) evaluation metrics that pertain to well-established functions and processes, will likely include measures previously developed, utilized and refined within individual trials networks, and DAIDS. Reuse of existing measures offers the potential to build on past experience, gain efficiency, establish baselines and, possibly, performance benchmarks. In other areas (e.g. cross-network coordination and collaboration) there may be little experience to draw upon, in which case new measures may need to be explored and tested for utility and feasibility.

The outcome of this participatory process will be a draft evaluation plan that describes the framework and logic model and associated potential measures, tools and resources and recommendations for piloting an initial cross-network evaluation system. With this type of design in place, evolving iterations of the system can be tested for feasibility and utility, and modified as needed.

Often, evaluations of research programs are done as one time, ad hoc events, with little precedent for systems approaches. (See for exceptions, the National Science Foundation's Engineering Research Centers [16] and the National Cancer Institute's Transdisciplinary Tobacco Use Research Centers (TTURCs) initiative [18,21]). In contrast, this effort takes a systems approach to evaluation, seeking to build rigorous evaluation into the funding process and lifecycle, so it is seamlessly integrated with other research activities and provides feedback at key points to inform decision-making at all levels. Thus, the final evaluation plan, when developed, will describe several key events and milestones that need to be addressed in a comprehensive evaluation system. This approach also recognizes that evaluation questions, resources and activities can change depending upon the stage of the initiative, demands upon scientific study, and many other operational and management factors, and as such, can inform a flexible evaluation system capable of responding to the kinds of shifts that take place in a research environment as dynamic is this one.

## Conclusion

The trend toward "big science" calls for the development of systems for managing and evaluating large scale, multi-site, collaborative scientific research initiatives. The need for evaluation systems is particularly great given increasing pressure for accountability for public program outcomes. Yet, large scale science has its own unique goals, context and requirements that must be taken into account when designing ways to evaluate success. An ideal scientific research enterprise has been defined as one that 1) invests in work that impacts significant social and scientific challenges and responds to new discoveries; 2) fosters a wide network of relationships that generates relevant questions, recognizes emerging issues, and sustains significant, cutting-edge programs of work; 3) develops and nurtures the human and organizational capacity to conduct research; and 4) recognizes and communicates its impact on the world [44]. To date, there a few examples to draw upon, which indicate how best to model the successful evaluation of this type of large scale research initiative. In this context, DAIDS' newly restructured clinical research networks are an example of a large research initiative seeking to proactively address multiple challenges as it adapts to meet the ideals of a successful research enterprise.

At the highest level, the findings from this process provide a 'global' view and insight into the network stakeholders' thoughts, views, and perceptions of the factors critical to the networks' success. The HIV/AIDS clinical research networks comprise thousands of individuals with a variety of skills and training, working in hundreds of different organizations, in almost 50 different countries. The diversity of expertise, interests, priorities and cultures represented in the networks, while vital to their strength, pose challenges to maintaining a sense of unity of purpose and teamwork. The findings reveal areas of the network that stakeholders see as mission critical, and the relative importance of these different areas. For example, we have learned, for the first time, that across the board, stakeholders believe that the biomedical objectives selected by the networks, are the most important factor for network success. This finding alone indicates that despite the diversity inherent in the networks, science is the primary driver. Given our interest in developing a comprehensive approach to the evaluation of the networks, this finding provides a data-driven basis for taking next steps, such as efforts to understand how the networks determine their biomedical objectives, how those objectives are vetted, how they align (or not) with those of other complementary efforts, how well they match (or not) the strengths and resources of the networks, and how successful have the networks been in reaching these objectives. A similar examination can occur across all clusters of success factors yielding a great deal of detailed information about what should be measured and how. Additionally, the findings allow us to see what other 'clusters' of success factors are seen by the stakeholders as being related, both conceptually and in their importance. For example, the findings reveal that scientific agenda setting (including prioritization) and relevance to study participants are both seen as being closely related to the biomedical objectives and nearly as important. This provides additional layers of understanding and again, suggests potential directions in which the evaluation system development can proceed, in direct alignment with what the networks themselves see as the most important activities for achieving success.

The findings from this process allow us to map, in even greater detail, the success factors at multiple levels (i.e. statement or cluster) to the demographics of the stakeholder participants. For instance, we can start to determine if within one area of science (e.g. HIV vaccines) certain success factors are seen as more important than they are viewed by stakeholders working in a different area, such as HIV therapy. Similarly, we can learn how stakeholders' time in the networks or their roles relates to their views of what is important for network success. This kind of information can help to reveal the basis of conflict or competition which could be creating obstacles (e.g. within networks, between networks or network collaborators) and point to activities which could be undertaken to mitigate such.

Taken together, these findings and the processes described here are the first of their kind relative to this large research initiative and provide valuable insights into the nature and functioning of the HIV/AIDS clinical research networks. The concept map and the logic model reflect evaluation criteria that account for the goals of multiple stakeholder groups and inform the first efforts to develop a systematic approach to evaluating this complex enterprise.

In this paper, we described a stakeholder-driven conceptual framework development process as an initial step in the creation of an evaluation system. This participatory activity engaged scientists, staff, and community advocates with first-hand experience of the initiative, in co-constructing the framework for an evaluation system. This stakeholder-driven process served two purposes. It was designed primarily as a prospective planning tool to identify the success factors of the coordinated clinical research networks and, therefore, elements of the evaluation system. It also enabled a "pulse check" on the extent to which stakeholders have embraced the goals and guiding principles embedded in the guiding principles behind the recent restructuring of the clinical trials networks.

Moreover, we detail how the results of the concept map relate to the goals of the networks, and point to how the map can potentially be used to guide the development of evaluation plans for this complex initiative. That said, the map can inform other 'views' or interests, for which an extensive background in HIV/AIDS clinical research would not be required. For example, for someone interested in researching the dynamics and complexities of team science, a contextual knowledge of that field could inform the interpretation of the HIV/AIDS network concept map (presented herein) in a very different way, and for a different purpose. In a sense, it depends on the 'lens' through which the user wishes to view the map, as this conceptualization activity could support multiple inquiries.

In the context of evaluation of large scale scientific research initiatives we found support for the idea that "big science" is accountable for a wider variety of processes, activities and outcomes than traditionally organized science research. This finding is consistent with other writing on this subject [3,5,19,20,45]. It is not sufficient to simply apply the same processes of evaluation traditionally associated with individual investigator awards because the expectations of large scale science are broader.

We also found that the sheer size and complexity of the initiative requires attention to a host of management challenges that would not be applicable to traditional individual investigator awards. The recent restructuring of the coordinated clinical research networks was undertaken to address many of these management issues, focusing on efficiency, coordination, responsiveness, capacity building and evaluation. This assessment indicated an awareness of these management issues among stakeholders, but revealed that overall, these stakeholders place more value on outcomes than the administrative and management processes required to achieve those outcomes.

Finally, we found a number of ideas and concepts that emerged from the concept map that were consistent with other evaluation planning efforts carried out in large scientific research initiatives. Concepts related to scientific outputs and goals, communication and collaboration, and management policies and procedures have appeared in other framework development efforts [17,21,32]. Similarities notwithstanding, the conceptualization process was unique to the DAIDS clinical research networks. Nonetheless, we believe that this work contributes to an emerging database of "big science" evaluation planning efforts where concept mapping was used to conceptualize elements for evaluation. As this collection of conceptualization projects increases, the potential for meta-analysis across participants, ideas, data (sorting and rating), and findings (maps and patterns) is possible.

Several limitations and caveats of this work are worth noting. First, the results of the development of a conceptual framework are context specific. Although several clusters that emerged from the concept mapping process were similar to the content found in other large scale research evaluation planning processes where concept mapping has been used, the ideas generated, organized and prioritized by the stakeholders were specific to the DAIDS clinical networks. Thus, while limiting the generalizability of the results, the use of concept mapping had the advantage of focusing the scope of the evaluation, increasing the potential for an efficient and sustained evaluation effort. Second, the results of the development of the conceptual framework for the evaluation are highly dependent upon informed and meaningful participation. While we sought to engage broad-based participation from those that have a stake in the processes and outcomes of the network activities, it is clear that some groups had greater numbers of participants than others. This is fairly typical in participatory evaluation planning. Concept mapping has several advantages for the management of participation, including multiple opportunities for engagement, multiple forms of input, and multiple products for interpretation.

Given the complexity, size, and scope of the activities of the clinical research networks, the time spent considering and engaging stakeholders in the conceptualization process is likely to yield benefits in later stages of the evaluation. Additionally, we used mean cluster ratings to represent the perspectives of the participants. This approach means that inferences about ratings must be made with caution. The primary intent of this project was to sample for heterogeneity to assure that a varied range of people had an opportunity to volunteer to participate and that no one who might want to take part would be excluded. While average ratings may represent well the views of the participants themselves and of the best represented subgroups of participants, it is not possible to say how well such averages represent groups that did not volunteer at comparable rates. In general, sampling issues in this context are a challenge and the opportunistic volunteer sampling used here was intended as a compromise between inclusion and representativeness. If subgroup comparisons are desired, we will incorporate a representative sampling approach.

Through this example, we've described an approach to a participatory conceptual framework development process as a part of evaluating a large scale clinical research endeavor. These approaches to planning and identifying key elements of an evaluation system may serve as useful guidance to others who are also pioneering in this emerging field.

## Competing interests

The authors declare they have no competing interests.

## Authors' contributions

All of the authors helped to conceptualize ideas, interpret findings, and review drafts of the manuscript. JK conceived of the study and drafted significant portions of the manuscript. MK contributed to the project design and interpretation of the results and provided project support and management at each step. KQ conducted the concept mapping analysis, the background literature review and contributed to the overall manuscript writing. SR assisted in revisions of the manuscript. WT provided guidance to the background research and project implementation. All authors approved the final manuscript.

## Supplementary Material

Additional file 1 **Statements organized by cluster with average importance ratings by statement and cluster**. Individual statements within concept map clusters, with mean importance ratings, by statement and cluster. Click here for file
