# Supplementary material for: Developing a conceptual framework for an evaluation system for the NIAID HIV/AIDS clinical trials networks
Source: Health Res Policy Syst. 2009 May 21;7:12. doi: 10.1186/1478-4505-7-12 (PMC2695433; doi:10.1186/1478-4505-7-12)
Supplement: Additional file 1 — Statements organized by cluster with average importance ratings by statement and cluster. Individual statements within concept map clusters, with mean importance ratings, by statement and cluster. [file 1478-4505-7-12-S1.doc]

| ***Cluster 1: DAIDS Policies and Procedures*** | | |
| --- | --- | --- |
| **72** | DAIDS policies reflect what is required for good science, protection of human subjects, and safety. | 4.4 |
| **25** | NIAID's budgeting process for the networks and the sites is transparent. | 4.2 |
| **88** | DAIDS streamlines their monitoring/ auditing procedures. | 4.03 |
| **44** | NIAID considers multiple factors in funding networks and sites. | 3.93 |
| **65** | DAIDS develops a clear policy about the purchase of study products (e.g. ARVs, vaccines, prophylactics). | 3.7 |
| **6** | DAIDS develops a clear policy that facilitates generic drug use. | 3.5 |
| **Average** | | **3.96** |
|  | | |
| ***Cluster 2: Operations and Management*** | | |
| **48** | protocols are closed out, analyzed and published in a timely manner. | 4.38 |
| **81** | DAIDS provides clear, consistent messages about goals, objectives and expectations. | 4.37 |
| **24** | there is a streamlined protocol development and implementation process. | 4.2 |
| **40** | efficient and reasonable regulatory processing can be established. | 4.16 |
| **14** | there is a performance-oriented network culture. | 3.98 |
| **8** | DAIDS has methods for managing complex endeavors. | 3.95 |
| **43** | standardized systems of accountability are integrated across networks and DAIDS. | 3.92 |
| **66** | clear direction is given on which DAIDS priorities are within the scope of the networks versus which DAIDS priorities should be achieved via other grant mechanisms. | 3.9 |
| **91** | there is a defined process for networks to use in reviewing future site expansion. | 3.74 |
| **18** | DAIDS medical officers/ program officers provide a consistent level of input/oversight to all networks. | 3.74 |
| **34** | there are uniform standards for site development. | 3.61 |
| **10** | the standard operating procedures across each of the networks are consistent. | 3.48 |
| **70** | DAIDS staff who work with the studies have a voice in decisions to approve proposals/ protocols. | 3.31 |
| **35** | each component of the network (committees, SDMC, labs, ops centers, sites) has the authority to perform its duties and responsibilities without the interference of the others. | 3.27 |
| **38** | a central IRB system is established. | 3 |
|  | **Average** | **3.8** |
|  | | |
| ***Cluster 3: Resource Utilization*** | | |
| **12** | there is accountability at all levels. | 4.51 |
| **87** | investigators have adequate qualifications and experience. | 4.37 |
| **9** | networks provide developing world sites with the clinical, regulatory, pharmacy, and laboratory support needed to ensure quality. | 4.3 |
| **69** | principal investigators demonstrate scientific leadership and innovation. | 4.26 |
| **41** | nonproductive activities are curtailed early. | 4.25 |
| **33** | investigators commit adequate time to network activities. | 4.18 |
| **21** | there is integrated use of domestic and international sites. | 3.94 |
| **49** | there is management of specimen flow (including from site to site-affiliate-lab to repository to end-point lab). | 3.91 |
| **56** | the administrative burden on sites is minimized. | 3.9 |
| **23** | there is expertise in infrastructure development. | 3.78 |
| **2** | there is consistent and equitable resource allocation within networks. | 3.74 |
| **71** | investigators are diverse, representing minority, international, female and/or young investigators. | 3.66 |
| **76** | the Core Laboratory is composed of Specialty Laboratories selected to provide requisite breadth of laboratory expertise to support the comprehensive agenda of the networks. | 3.62 |
| **28** | CTUs can implement protocols from different priority areas. | 3.6 |
|  | **Average** | **4** |
|  | | |
| ***Cluster 4: Community Involvement*** | | |
| **42** | the dignity and human rights of participants are respected. | 4.69 |
| **52** | sites have the scientific and technical skills needed to pursue the research agenda. | 4.42 |
| **84** | clinical trial sites successfully meet recruitment and retention goals | 4.27 |
| **74** | research is conducted acknowledging the culture, norms and values of the community they are working with. | 4.17 |
| **39** | consideration is given to the differences in conditions in resource poor nations. | 3.94 |
| **83** | community support, training and education are provided. | 3.91 |
| **31** | appropriate and relevant community representation is included at all levels; institute, network, and site. | 3.85 |
| **77** | ethics, community and behavioral teams provide input early in protocol development. | 3.8 |
| **80** | research sites provide hours to make participation accessible to subjects. | 3.75 |
| **68** | the community in included in every stage of a protocol. | 3.67 |
|  | **Average** | **4.05** |
|  | | |
| ***Cluster 5: Collaboration, Communication, Harmonization*** | | |
| **58** | there is communication within each network. | 4.36 |
| **54** | networks are cost effective in getting studies accomplished. | 4.1 |
| **3** | leadership and trial sites are consulted to identify areas that will benefit from harmonization and closer collaboration (such as laboratory SOPs, data management, training, etc.), but are supported to address the needs that are unique to the network. | 4.06 |
| **90** | there is collaboration within and among the networks. | 4.04 |
| **67** | information, resources and materials are shared across networks. | 3.93 |
| **1** | standardize key laboratory procedures across the networks. | 3.86 |
| **75** | there is regular and frequent communication among clinical research networks. | 3.85 |
| **55** | the vision and goals are shared. | 3.85 |
| **29** | there is worldwide collaboration. | 3.83 |
| **51** | networks develop and support mutually beneficial coordination among the networks and DAIDS and other relevant NIH networks, federal agencies and NGO research organizations. | 3.78 |
| **36** | networks harmonize key training, laboratory, network evaluation, data management, and other key functions across networks. | 3.7 |
| **45** | effective cross network training is utilized. | 3.69 |
| **53** | networks develop a harmonized data management system. | 3.61 |
| **59** | annual and biannual meetings of the networks overlap to allow for sharing of scientific data and future planning of the scientific agenda of each group. | 3.57 |
| **32** | public-private partnerships with industry collaborators are used. | 3.56 |
| **26** | there are standardized tool kits for use in behavioral studies. | 3.22 |
|  | **Average** | **3.81** |
|  | | |
| ***Cluster 6: Scientific Agenda Setting*** | | |
| **7** | networks develop protocols with attainable goals. | 4.49 |
| **78** | the proposed scientific priorities and research plan is feasible. | 4.48 |
| **63** | networks reassess and reprioritize their scientific priorities as the field evolves. | 4.47 |
| **64** | networks focus on high priority trials that will not be done in the private sector. | 4.18 |
| **16** | there is communication and cooperation between stakeholders in the planning of science. | 4.08 |
| **19** | there is acknowledgement of and support for the scientific contributions of international research partners. | 4.08 |
| **4** | networks obtain scientific input and involve a large group of clinical investigators in the research agenda. | 3.88 |
| **62** | networks focus on complementary pieces of the research agenda. | 3.78 |
| **11** | there is collaboration with experts outside the networks. | 3.75 |
| **57** | networks integrate biomedical and technological advances with behavioral intervention strategies. | 3.39 |
|  | **Average** | **4.06** |
|  | | |
| ***Cluster 7: Biomedical Objectives*** | | |
| **82** | networks produce high-quality, scientifically valid results. | 4.74 |
| **5** | networks produce results that inform the way in which HIV infection is treated and prevented. | 4.59 |
| **20** | networks focus on answering the top scientific questions related to the treatment and prevention of HIV and its complications. | 4.5 |
| **60** | scientific results are published and disseminated widely. | 4.43 |
| **86** | morbidity (rate of occurrence) of HIV/AIDS is reduced or eliminated. | 4.37 |
| **85** | mortality (death rate) from HIV/AIDS is reduced or eliminated. | 4.34 |
| **89** | research offers the potential to find practical strategies for HIV prevention that can be applied quickly. | 4.06 |
| **50** | evaluate vaccines for the prevention of HIV sexual transmission among populations at risk. | 3.95 |
| **17** | the development of antiretroviral (ARV) resistance is minimized. | 3.93 |
| **61** | the understanding of HIV-1 pathogenesis is furthered. | 3.91 |
| **22** | criteria for the advancement of the most efficacious and least toxic products are developed. | 3.77 |
| **37** | networks assess research issues and questions in the context of prevention and treatment policies. | 3.71 |
| **73** | substudies add value to the experimental design of the parent protocols. | 3.68 |
| **79** | a safe and at least partially effective microbicide is identified. | 3.51 |
|  | **Average** | **4.11** |
|  | | |
| ***Cluster 8: Relevance to Participants*** | | |
| **46** | high impact research results get translated into practice. | 4.48 |
| **15** | the questions to be addressed are relevant to the country/populations in which the study is done. | 4.43 |
| **30** | populations at greatest risk for HIV/AIDS are adequately represented in clinical research. | 4.21 |
| **27** | networks consider the gender, ethnicity, socioeconomic status, and other demographic characteristics of the target population during planning. | 3.86 |
| **13** | research is appropriately integrated with clinical and community service delivery. | 3.81 |
| **47** | treatments for HIV-infected people in special populations are evaluated. | 3.73 |
|  | **Average** | **4.09** |
